# DOLD-Net: Dense occluded livestock detection via global-local feature collaboration

**DOI:** 10.1371/journal.pone.0356771

**Published:** 2026-08-25

**Authors:** Kaida Jia, Yang Chai, Baozhou Chen, Zunhai Gao

**Affiliations:** School of Mathematics and Computer Science, Wuhan Polytechnic University, Wuhan, HuBei, China; Chongqing Medical University, CHINA

## Abstract

Precise livestock detection is fundamental to smart agriculture; however, real-world environments often present challenges such as high object density, severe occlusion, and ambiguous boundaries. To address feature integrity degradation under such conditions, this paper proposes DOLD-Net: a model for densely occluded livestock detection utilizing global–local feature collaboration. First, this paper develops a Dual-Branch Occlusion-Aware Network (DBOAN) backbone to mitigate feature confusion stemming from object aggregation. The DBOAN models both local gradient flows and global topological structures. By integrating these two aspects, the network preserves fine-grained details while maintaining coherent spatial relationships. To further resolve semantic discontinuities in occluded regions, a Context-Guided Focus Propagation (CGFP) mechanism is designed. The CGFP constructs a global core representation through long-range dependency modeling. This representation propagates discriminative semantic priors in a top-down manner across feature hierarchies. Furthermore, a Frequency–Spatial Anti-occlusion Module (FSAM) decouples entangled representations in the frequency domain to sharpen ambiguous boundaries. Additionally, a Context-Aware Occlusion Fusion Module (CAOFM) compensates for local information loss through adaptive cross-scale feature interactions. Extensive experiments on four public datasets demonstrate that DOLD-Net achieves a superior balance between accuracy and efficiency, attaining AP scores of 57.7%, 67.5%, 52.5%, and 62.5%, respectively. The source code is publicly available at https://github.com/Jiakaida/DOLD-Net.

## Introduction

Accurate livestock detection is a pivotal supporting technology within smart agriculture systems, essential for optimizing farm management decisions, disease prevention, early-warning protocols, and resource allocation [1]. Global demand for animal products continues to grow. Meanwhile, increasing societal attention to environmental sustainability and animal welfare standards is reshaping the livestock industry. As a result, the industry is transitioning toward efficient and sustainable intelligent management models. In recent years, computer vision has emerged as a key enabling technology for achieving precise livestock management [2–4]. The vision-based non-contact monitoring paradigm offers significant advantages. Specifically, it effectively mitigates animal stress responses induced by human intervention, thereby improving animal welfare. Furthermore, this approach can be integrated with automated monitoring systems. It enables real-time perception and intelligent decision-making within the production process, thereby significantly improving efficiency and resource utilization.

However, in practical farming scenarios, factors such as dense aggregation, severe inter-individual occlusions, complex backgrounds, and variable postures present significant challenges for robust target detection. Conventional detection methods—including Histogram of Oriented Gradients [5], the Gaussian Mixture Model [6], and Deformable Part Models [7]—primarily relied on handcrafted feature extractors. These approaches struggle to adapt to random appearance variations in complex environments. In addition, they exhibit limited efficacy in handling severe occlusions and densely distributed targets.

In recent years, architectures based on Convolutional Neural Networks (CNNs) and Transformers [8] have yielded substantial advancements in object detection. Prominent frameworks, including two-stage detectors (e.g., the R-CNN series [9]), single-stage detectors (e.g., the YOLO series [10]), and Transformer-based models (e.g., DETR [11]), have significantly advanced livestock detection capabilities. Guo et al. [12] enhanced pig re-identification by using a weighted association algorithm that combines IoU distance with appearance embeddings. Noe et al. [13] proposed an instance segmentation approach based on the Mask R-CNN framework and further incorporated a lightweight object tracking algorithm as a post-processing module to enable automatic detection and continuous tracking of cattle mounting behaviors. Tian et al. [14] proposed GCS-MUL, a lightweight CNN predicated on multi-task learning. The architecture integrates a GhostConv-based backbone with the CBAM attention mechanism. It utilizes a segmentation head to achieve high-precision, real-time detection and semantic segmentation of heterogeneous targets in dairy farms. However, during collective feeding, head occlusions often cause multiple instances to be detected as a single target, thereby increasing the missed detection rate. Mar et al. [15] proposed a vision-based cattle detection and tracking framework that integrates instance segmentation with multi-feature association. By combining spatial location, color, texture, and deep appearance features, this method improves the robustness of multi-cow tracking under complex farm environments. Xiao et al. [16] developed DHSW-YOLO for monitoring duck group behavior, achieving a 94.4% mAP. However, the method was validated only under two fixed lighting conditions and does not consider dynamic natural light variations. Similarly, Guo et al. [3] developed YOLO-SDD, which integrated wavelet-enhanced convolution and an occlusion-aware attention mechanism to achieve an 84.27% AP under dense occlusion. Despite these gains, its high computational complexity precludes efficient deployment on computationally limited edge devices. Furthermore, Li et al. [17] introduced EMSC-DETR, leveraging SDTM and CoT modules for multi-scale chicken detection in complex environments. Although the model shows robust generalization, it still has a high parameter count and computational overhead, limiting its deployment.

Notable progress has been achieved in livestock detection within dense environments. However, substantial challenges still persist. These challenges are particularly evident in scenarios with severe occlusion and dense aggregation. Existing methods often enhance performance by stacking increasingly complex modules. However, this strategy significantly increases model parameters and computational overhead, which hinders large-scale deployment in real-world applications. To balance the trade-off between model complexity and detection accuracy, this paper proposes the Dense Occluded Livestock Detection Network (DOLD-Net). DOLD-Net employs a progressive “global-to-local” refinement paradigm to systematically address challenges arising from occlusion and aggregation. The proposed architecture comprises three tightly coupled stages: the Backbone, Neck, and Head. To facilitate robust feature extraction, this paper develops a Dual-Branch Occlusion-Aware Network (DBOAN). Recognizing that single-view features are insufficient for discriminating crowded objects, the DBOAN establishes a collaborative learning mechanism integrating local gradient flows with global topological modeling. This synergy provides a robust feature foundation that effectively separates overlapping instances during the initial stages of representation learning. To bridge the semantic gaps caused by occlusion, a Context-Guided Focus Propagation (CGFP) framework is designed in the Neck stage. Different from simple feature aggregation, the CGFP injects global semantics to guide local feature restoration. Specifically, a Cross-Scale Feature Enhancer (CSFE) extracts key semantic cues to build a global core prior. This prior provides top-down guidance, enabling the inference of occluded targets using global context. A Frequency–Spatial Anti-occlusion Module (FSAM) is integrated into this propagation pathway to address boundary blurring within dense clusters. The FSAM incorporates frequency-domain analysis to decouple entangled features. This process sharpens distinctions between overlapping objects that may be missed by purely spatial modeling. Finally, to ensure cross-scale feature consistency, this paper proposes a Context-Aware Occlusion Fusion Module (CAOFM). By exploiting contextual complementarity between adjacent feature levels via pairwise interactions, the CAOFM recovers missing feature details. It also repairs spatial discontinuities in the features. To translate these refined features into accurate predictions, the D-FINE [18] architecture is employed as the detection head. The detection head reformulates bounding box regression as an iterative optimization process over probability distributions. It also incorporates global optimal localization self-distillation. This design ensures that enhanced features are mapped to precise bounding boxes with high accuracy and minimal training overhead.

**Fig 1:**
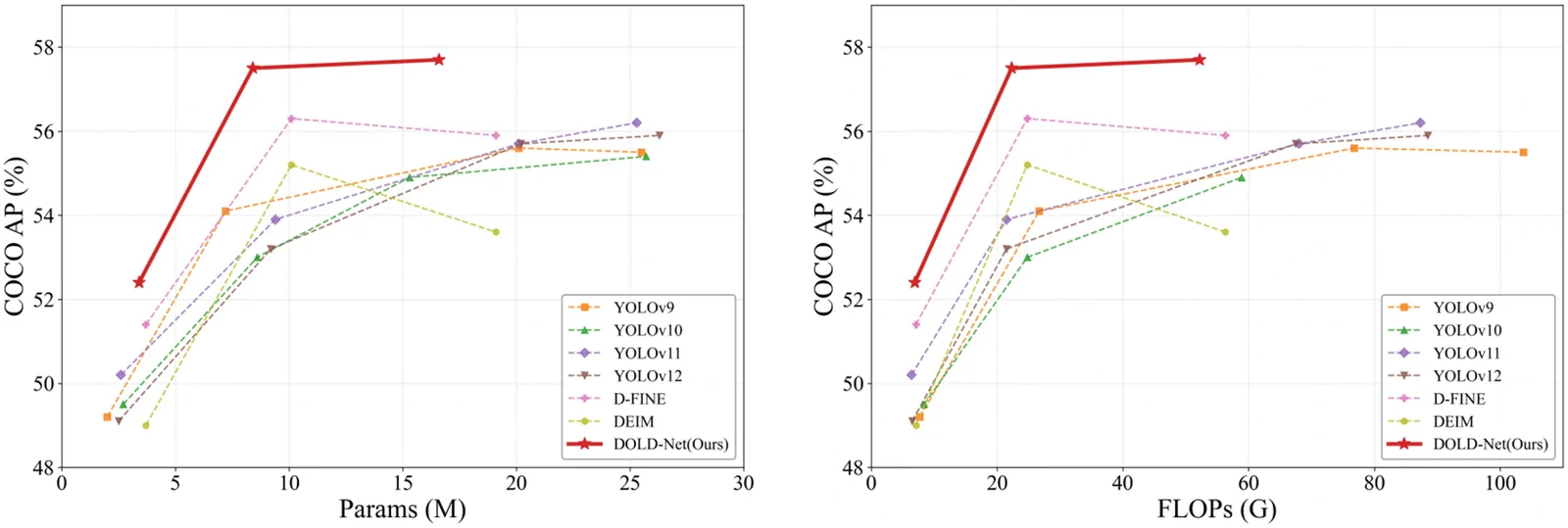
Comparison of parameters and cost of different detection models. (a) Model Parameters. (b) Computational Cost (FLOPs).

This study validated the effectiveness of DOLD-Net using four publicly available livestock detection datasets. Experimental results showed that DOLD-Net achieved *AP* scores of 57.7%, 67.5%, 52.2%, and 62.5% on the four datasets, surpassing state-of-the-art methods. Fig 1 compares different models in terms of parameter count, computational cost, and model accuracy. Meanwhile, the N variant achieves a lightweight design with only 6.9 GFLOPs and 3.4 M parameters. It maintains competitive performance, demonstrating significant potential for practical application.

## Materials and methods

### Dataset

To validate the effectiveness of the DOLD-Net in handling occlusion and dense targets, this work conducted extensive experimental evaluations across four public datasets: GooseDetect [19], SheepCounter, CherryChèvre [20], and ChickenFlow [3]. These datasets encompass diverse challenges, including varying scenes, lighting conditions, and livestock species. They facilitate a comprehensive assessment of the model’s robustness and generalization capabilities for dense occlusion livestock detection.

The GooseDetect dataset consists of two subsets: one containing only adult geese ($GooseDetect_{b}^{lion}$) and the other focusing on goslings ($GooseDetect_{s}^{lion}$). This dataset is characterized by severe occlusion and high-density environments. Daytime and nighttime lighting variations further pose challenges to accurate detection. The SheepCounter dataset was collected from an aerial perspective using UAVs. Occlusion is relatively moderate in this dataset. Nevertheless, the top-down perspective produces many small targets, which requires the model to perform precise small object detection. Moreover, the ChickenFlow dataset presents severe occlusion and density-related problems. Rapid movement of chickens introduces motion blur, further increasing detection challenges. In contrast, the CherryChèvre dataset features moderate occlusion. It also exhibits significant environmental variability. This variability includes domain shifts between indoor and outdoor scenes, complex backgrounds, and varying illumination conditions. Additionally, significant variations in coat color among individual goats further augment the detection difficulty. The diversity of these four datasets ensures rigorous validation across a wide range of real-world scenarios.

### Model of DOLD-Net

**Fig 2:**
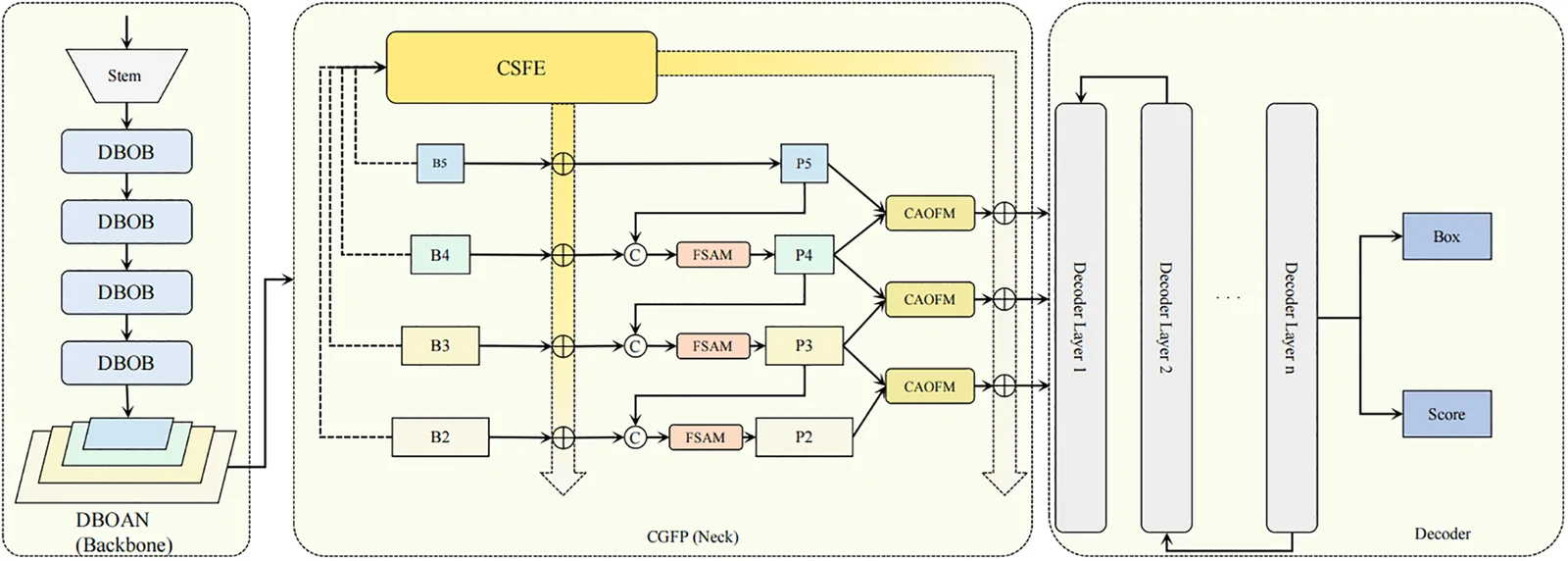
The structure of the proposed DOLD-Net model. The DBOAN backbone extracts hierarchical multi-scale features (B2–B5), the CGFP integrates and fuses them through the CSFE, FSAM, and CAOFM to generate the feature pyramid (P2–P5), and the Decoder performs object classification and bounding box prediction.

To address the issues of detail loss and semantic discontinuity in dense livestock detection, this paper proposes DOLD-Net. As shown in Fig 2, the model is built upon the principle of collaborative perception and progressive restoration. Specifically, the model aims to mitigate occlusion effects through a three-stage process. First, the DBOAN backbone establishes a robust feature representation by collaboratively encoding local texture details and global topological structures. Second, the CGFP framework serves as the core restoration engine. A global semantic prior is first constructed via CSFE, and the boundaries of occluded objects are subsequently refined in the frequency domain through FSAM. It also repairs cross-scale feature inconsistencies using CAOFM. Finally, the D-FINE head transforms these refined features into precise probability distributions for accurate final detection.

### DBOAN

**Fig 3:**
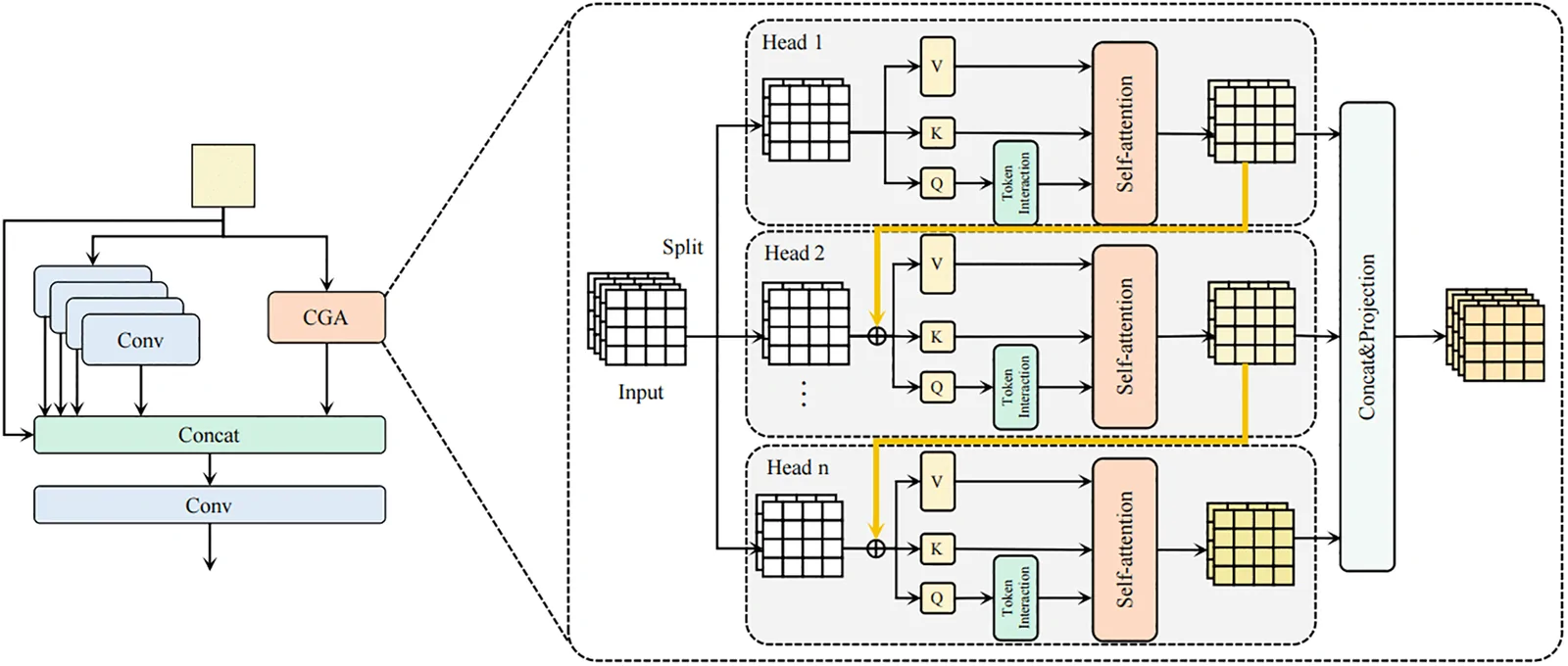
The structure of DBOB.

In dense and occluded livestock detection, conventional backbone networks encounter significant limitations. First, livestock typically exhibit strong morphological similarity within the same class. Therefore, differentiating individual boundaries solely through stacked convolutional layers is difficult under dense aggregation. This results in feature confusion. Second, severe occlusion induces significant feature degradation. Consequently, the model must infer complete object structures from fragmented local cues. To address these bottlenecks, this paper proposes the DBOAN architecture. The DBOAN comprises a stem and multiple Double-Branch Occlusion-Aware Blocks (DBOB). In the stem stage, this paper adopts a structure consistent with Hgnetv2 [21] for initial feature extraction. The extracted feature maps are subsequently processed by multiple DBOB to model occlusion structures at different scales. This process generates a multi-scale feature representation. The detailed configuration is shown in Fig 3.

The local branch of the DBOAN partitions the gradient tensor into non-overlapping channels. These feature maps are processed through diverse convolutional blocks. This architecture ensures that each intermediate feature map is supervised by independent gradient signals. Specifically, it establishes *n* parallel backpropagation pathways extending to the input layer. In this configuration, the direct concatenation path mitigates gradient vanishing, while the pathways through convolutional layers supply additional gradient components. This multi-path gradient flow mechanism facilitates optimization stability and strengthens the discriminative capacity of local representations.

The global branch captures target geometry and spatial relationships, enabling effective separation of densely occluded instances. Although transformers excel at capturing long-range dependencies, they incur high computational complexity. Moreover, in multi-head attention, individual heads frequently learn similar linear projections, resulting in significant redundancy within the attention maps. Cascaded Group Attention (CGA) [22] decomposes the computation of each attention head by feeding distinct feature subsets. This approach effectively mitigates computational redundancy inherent in the multi-head attention mechanism. Additionally, CGA augments the model’s representational capacity by facilitating increased network depth. Consequently, this paper incorporates CGA within the global branch to minimize feature redundancy. The attention formula for this mechanism is given by Eq. (1).


$$\begin{array}{c} {\tilde{X}}_{ij}=Attention(X_{ij}W_{ij}^{Q},X_{ij}W_{ij}^{K},X_{ij}W_{ij}^{V}),\;{\tilde{X}}_{i+1}=Concat[{\tilde{X}}_{ij}{]}_{j=1:h}W_{i}^{P}, \end{array}$$ (1)

where *h* represents the total number of heads, and $W_{ij}^{Q}$, $W_{ij}^{K}$, and $W_{ij}^{V}$ are projection layers that map the input feature segments to different subspaces. $W_{i}^{P}$ is a linear layer that projects the concatenated output features back to a dimension that matches the input dimension. Furthermore, the cascaded structure encourages the *Q*, *K*, and *V* layers to learn projections on features with richer information, thus enhancing the expressiveness of the attention mechanism. The mathematical expression for this mechanism is given by Eq. (2).


$$\begin{array}{c} X_{ij}^{\prime }=X_{ij}+{\tilde{X}}_{i(j-1)},\;1<j\leq h. \end{array}$$ (2)

In summary, the DBOAN enhances feature representation in complex occlusion scenarios through the integration of local and global modeling. The local branch facilitates stable extraction of fine-grained details through a multi-path gradient flow mechanism. Concurrently, the global branch employs CGA to reduce computational redundancy by decomposing feature interactions in multi-head attention. This enhances the model’s capacity to capture long-range dependencies and spatial relationships between targets. The synergy of the two branches enables DBOAN to accurately represent and separate densely occluded targets while maintaining computational efficiency.

### CGFP

To effectively mitigate semantic fragmentation and detail loss caused by severe occlusion, this paper develops the CGFP framework. Unlike conventional feature aggregation strategies, the CGFP functions as a systematic feature restoration module. Specifically, to mitigate the limited receptive field of local features, the CSFE constructs a comprehensive global core representation. This representation acts as a semantic prior, providing essential global context to the feature pyramid through a top-down propagation mechanism. This enables the model to effectively infer occluded target regions. Subsequently, the FSAM leverages the global context from CSFE and the robust representations from DBOAN to decouple densely adjacent object boundaries through frequency-domain analysis. Finally, the CAOFM is employed to exploit the complementary features provided by adjacent feature maps. By exploiting the inherent complementarity between adjacent feature levels, the CAOFM repairs spatial discontinuities and bridges semantic gaps, thereby yielding robust feature representations for the final prediction stage.

#### CSFE.

**Fig 4:**
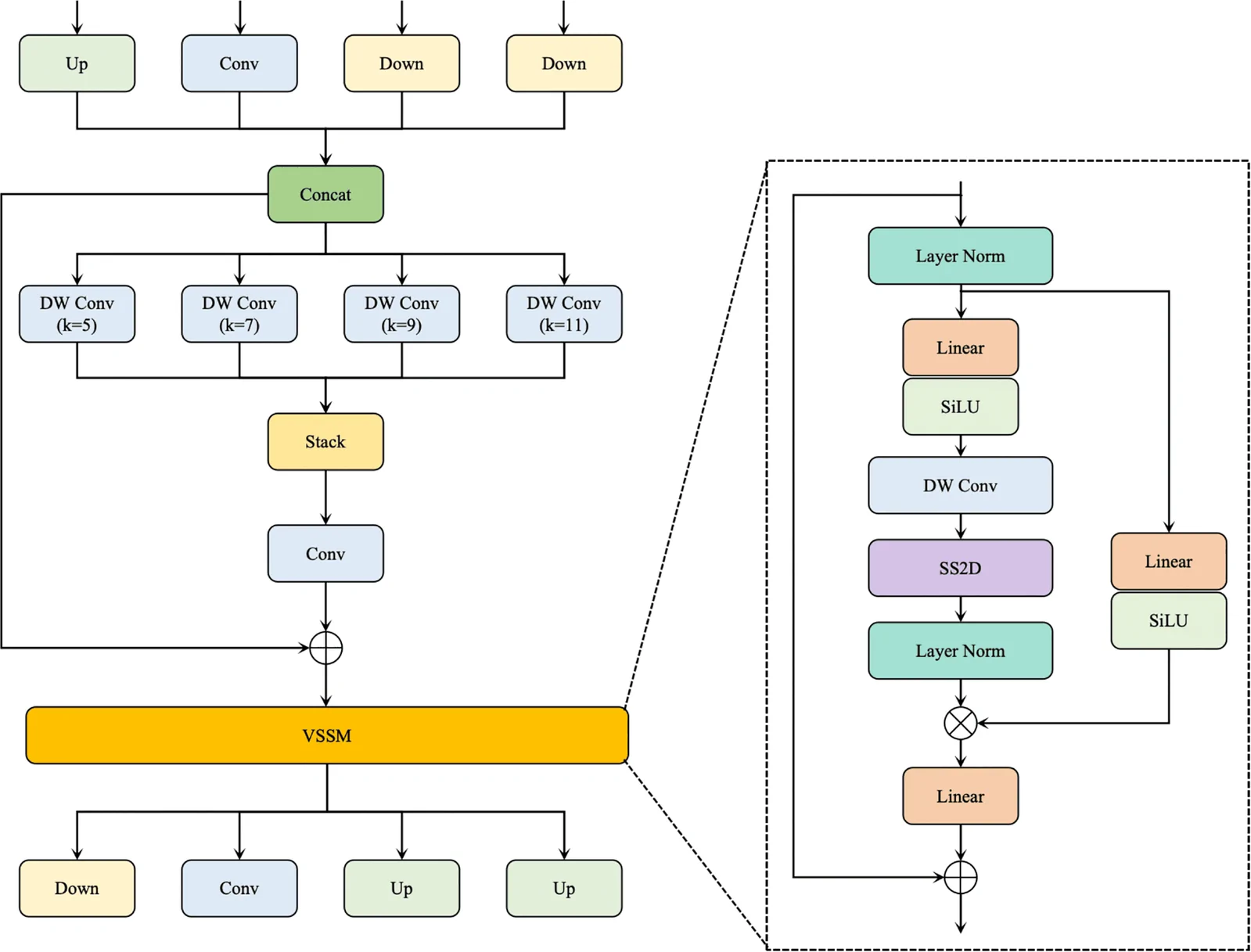
The structure of CSFE.

Standard object detectors, such as YOLO and DETR families, predominantly employ FPN-based architectures for multi-level feature fusion. However, this layer-by-layer information propagation paradigm has a critical limitation in dense livestock detection. Specifically, it cannot construct feature representations that capture the global semantic context explicitly. In FPNs, inter-layer interactions are indirect. Moreover, information from distant layers is susceptible to degradation during propagation, which hinders the formation of an effective global receptive field [23]. Due to the absence of a global context, the model must rely on the unoccluded local information to infer targets. However, these unoccluded regions often provide limited cues in densely populated scenes. To address this limitation, this paper proposes the Context-Scale Feature Enhancer (CSFE). Rather than passively aggregating features, the CSFE is designed to actively generate a comprehensive global core feature representation. This unified representation serves as a semantic prior, establishing a strong global contextual foundation that is subsequently injected across all feature scales. By employing a global-to-local reasoning mechanism, CSFE enables the model to infer missing target details when occlusion degrades local features. This inference is guided by the overall contextual distribution of the livestock, which ensures robust perception in challenging scenes. The architecture of the CSFE is shown in Fig 4.

Let $X=x_{0},x_{1},x_{2},x_{3}$ be the input set of four feature maps, where $x_{i}\in {\mathbb{R}}^{C_{i}\times H_{i}\times W_{i}}$. The first step performs channel mapping and scale alignment across the layers, as shown in Eq. (3).


$$\begin{array}{cc} {\tilde{x}}_{0} & ={Conv}_{1}(Upsample(x_{0})),\\ {\tilde{x}}_{1} & ={Conv}_{1}(x_{1}),\\ {\tilde{x}}_{2} & ={Conv}_{3}(x_{2},s=2),\\ {\tilde{x}}_{3} & ={Conv}_{3}(x_{3},s=4), \end{array}$$ (3)

where ${Conv}_{k}(\cdot )$ denotes a convolution operation with a kernel size of *k*. These operations unify the spatial size and channel dimension of feature maps across different scales. Subsequently, the aligned feature maps are concatenated as $X_{f}=Concat[{\tilde{x}}_{0},{\tilde{x}}_{1},{\tilde{x}}_{2},{\tilde{x}}_{3}]\in {\mathbb{R}}^{4h\times H\times W}$. To fully leverage the semantic information across different scales, a multi-scale depthwise separable convolution is employed to expand the receptive field of the fused feature $X_{f}$. This operation can be expressed as $Y_{i}={DWConv}_{k_{i}}(X_{f})$. Furthermore, residual connections and 1×1 convolutions are applied to enhance feature propagation while further fusing the different features. Finally, a Visual State Space Model (VSSM) [24] is utilized to construct a global core feature representation.

The core mechanism of VSSM is rooted in State Space Models (SSM). Traditionally, SSM are employed to represent linear time-invariant systems [25], effectively mapping one-dimensional input sequences to output sequences via latent hidden states to capture temporal dynamic relationships. Recently, the Mamba architecture [26] has successfully leveraged SSM for long-sequence modeling, demonstrating superior representation capabilities while maintaining linear time complexity.

**Fig 5:**
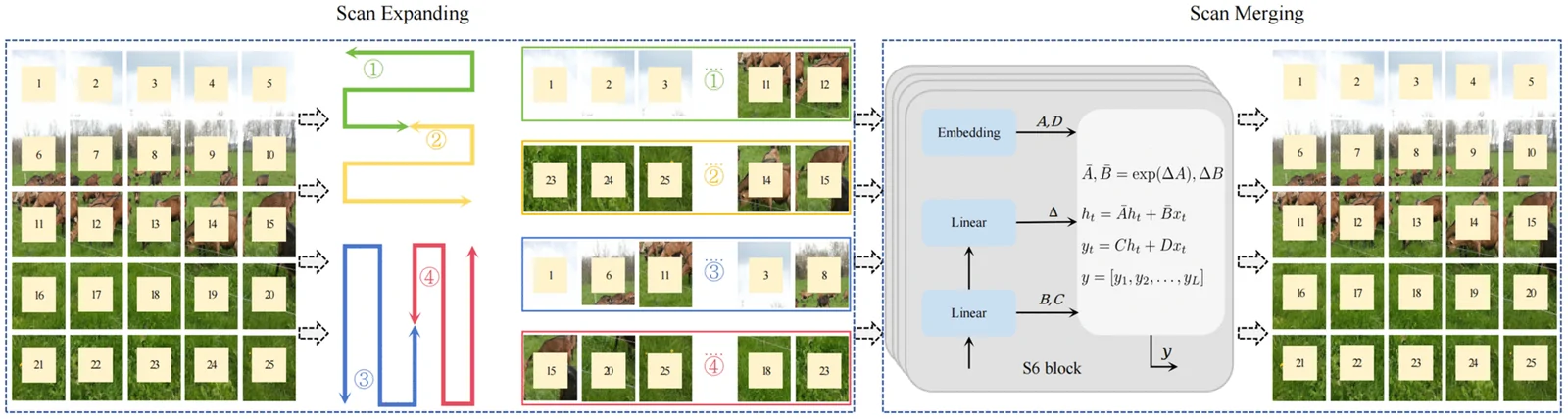
The structure of SS2D. Image sourced from [20] under the French Open License 2.0 license.

However, the inherent differences between two-dimensional (2D) visual data and one-dimensional (1D) sequence data hinder the direct application of the Mamba model to visual tasks. Visual tasks rely on 2D spatial information. Due to its limited receptive field, traditional 1D sequence modeling is insufficient for capturing latent dependencies in the spatial domain. To address this issue, the Visual State Space Model (VSSM) [24] introduces a two dimensional selective scanning mechanism (SS2D) as its core module, and Fig 5 shows its architecture. SS2D first extends the image blocks in four directions to generate four independent sequences. This multi-directional scanning strategy allows each element in the feature map to integrate global information from different directions. In this way, a global receptive field is constructed while maintaining linear computational complexity. After processing by the Selective Scanning State Space Model (S6), the feature sequences are merged back into a 2D feature map via a scanning merge operation, enabling effective visual modeling. The output features of SS2D are given by the Eq. (4).


$$\begin{array}{cc} & z_{i}=expand(z,i),\\ & {\bar{z}}_{i}=S6(z_{i}),\\ & \bar{z}=merge({\bar{z}}_{1},{\bar{z}}_{2},{\bar{z}}_{3},{\bar{z}}_{4}), \end{array}$$ (4)

where i∈[1,2,3,4] represents one of the four scanning directions, and the functions *expand*() and *merge*() correspond to the scanning expansion and scanning merge operations, respectively. The S6 block is the core VSSM operator in SS2D, facilitating interactions between each element and any previously scanned sample through a hidden state.

#### FSAM.

**Fig 6:**
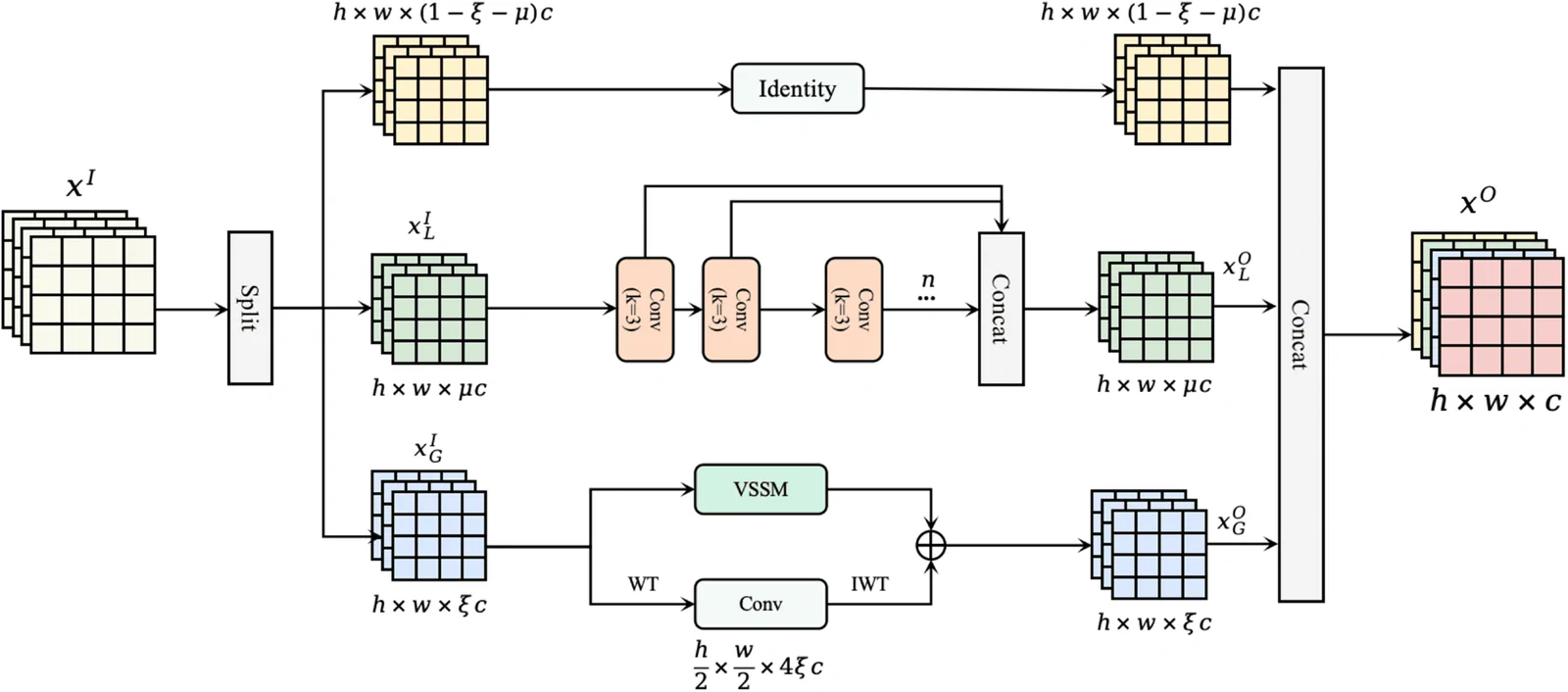
The structure of FSAM.

Following global semantic injection by the CSFE, an unresolved challenge remains: spatial feature entanglement. In high-density regions, adjacent livestock often exhibit ambiguous boundaries and similar texture. This characteristic limits the ability of spatial convolutions to discriminate between individual instances. To address this limitation, the FSAM is proposed to enhance boundary delineation and feature separation through a dual-domain analysis strategy, as shown in Fig 6. Specifically, the frequency-decoupling branch integrates discrete wavelet transforms with the VSSM to extract high-frequency edge information while preserving contextual consistency. Concurrently, the spatial branch leverages local convolutions to retain fine-grained details. Meanwhile, the redundancy-control branch employs identity mapping to preserve feature integrity. This design also helps minimize computational overhead. This multi-branch architecture separates densely occluded targets for precise detection.

The long-range wavelet transformation enhances VSSM by effectively distinguishing the boundaries of tightly adjacent individuals. The wavelet transform decomposes the features into low-frequency and high-frequency components. Among different wavelet families, the Haar wavelet is adopted because its shortest support preserves sharp local intensity transitions with minimal smoothing, making it more suitable for maintaining object boundaries in densely occluded livestock scenes than higher-order wavelets. The low-frequency component preserves the overall spatial layout and structural information of the livestock distribution, whereas the high-frequency component captures fine-grained boundary details. At the same time, VSSM is used to learn the global information representation. For the input feature map $x_{G}^{I}\in {\mathbb{R}}^{h\times w\times \xi c}$, where ξ represents the channel scaling factor, it is first transformed to obtain the feature map $x_{w}^{I}\in {\mathbb{R}}^{h\times w\times \xi c}$. In the FSAM module, the Haar wavelet transform first decomposes the input feature into one low-frequency sub-band $x_{LL}$ and three high-frequency sub-bands, namely $x_{LH}$, $x_{HL}$, and $x_{HH}$. These high-frequency sub-bands explicitly capture horizontal, vertical, and diagonal structural details. These four sub-bands are concatenated along the channel dimension to form $x_{cat}\in {\mathbb{R}}^{\frac{h}{2}\times \frac{w}{2}\times 4\xi c}$. Subsequently, a convolution is introduced for spatial modeling and feature fusion. To explicitly prioritize boundary sharpening, an independent, learnable channel-wise scaling factor γ is applied to adaptively recalibrate the response of each channel. Finally, the feature map is restored to its original spatial resolution through the Inverse Wavelet Transform (IWT), yielding $x_{w}^{O}\in {\mathbb{R}}^{h\times w\times \xi c}$, as shown in Eq. (5).


$$\begin{array}{c} (x_{LL},x_{LH},x_{HL},x_{HH})=WT(x_{w}^{I}),\;x_{cat}=Concat(x_{LL},x_{LH},x_{HL},x_{HH}),\;{\tilde{x}}_{cat}=\gamma ⊙Conv(x_{cat}),\;x_{w}^{O}=IWT({\tilde{x}}_{cat}). \end{array}$$ (5)

where Concat(·) denotes concatenation along the channel dimension, ⊙ represents element-wise multiplication along the channel dimension, and $\gamma \in {\mathbb{R}}^{4\xi c\times 1\times 1}$ is the learnable scaling vector.

Similarly, for the feature map $x_{G}^{I}\in {\mathbb{R}}^{h\times w\times \xi c}$, VSSM is used to extract global information, *i.e.*, $x_{m}^{O}=VSSM(x_{G}^{I})$. The final output feature map of this part is obtained by adding the output of the VSSM module to the output of the local information branch, as shown in Eq. (6). The resulting feature map is denoted as $x_{G}^{O}\in {\mathbb{R}}^{h\times w\times \xi c}$.


$$\begin{array}{c} x_{G}^{O}=x_{m}^{O}+x_{w}^{O}. \end{array}$$ (6)

The cascade of local convolution blocks enhances the ability to extract local features. The remaining feature map $x_{L}^{I}\in {\mathbb{R}}^{h\times w\times \mu c}$, where μ≤1−ξ, is processed through multiple cascaded convolutional blocks for local feature extraction. Finally, the outputs from each convolutional block are concatenated to produce the output feature map $x_{L}^{O}\in {\mathbb{R}}^{h\times w\times \mu c}$, as shown in Eq. (7)


$$\begin{array}{c} x_{Lj}^{O}=Conv\left(x_{Lj}^{I}\right),j\in 1,...,n,\;x_{L}^{O}=Concat([x_{L1}^{O},...,x_{Ln}^{O}],dim=-1). \end{array}$$ (7)

Finally, to address feature redundancy in high dimensional space, the residual part adopts identity mapping in this work. This approach minimizes unnecessary computational overhead while enhancing computational efficiency. Therefore, the final output of the FSAM module can be represented as shown in Eq. (8).


$$\begin{array}{c} x^{O}=Concat(x_{G}^{O},x_{L}^{O},x^{I}[(1-\xi -\mu )c:]). \end{array}$$ (8)

The FSAM utilizes a three-branch parallel architecture to facilitate deep synergy between the frequency and spatial domains. The wavelet-enhanced VSSM branch sharpens ambiguous boundaries of overlapping targets through frequency-domain decomposition. This design overcomes the limitations of spatial convolutions. Concurrently, the local convolution and identity mapping branches retain fine-grained details while preserving feature consistency. This multi-domain design separates dense clusters into separable instances, generating boundary-aware features for fusion while maintaining computational efficiency.

#### CAOFM.

**Fig 7:**
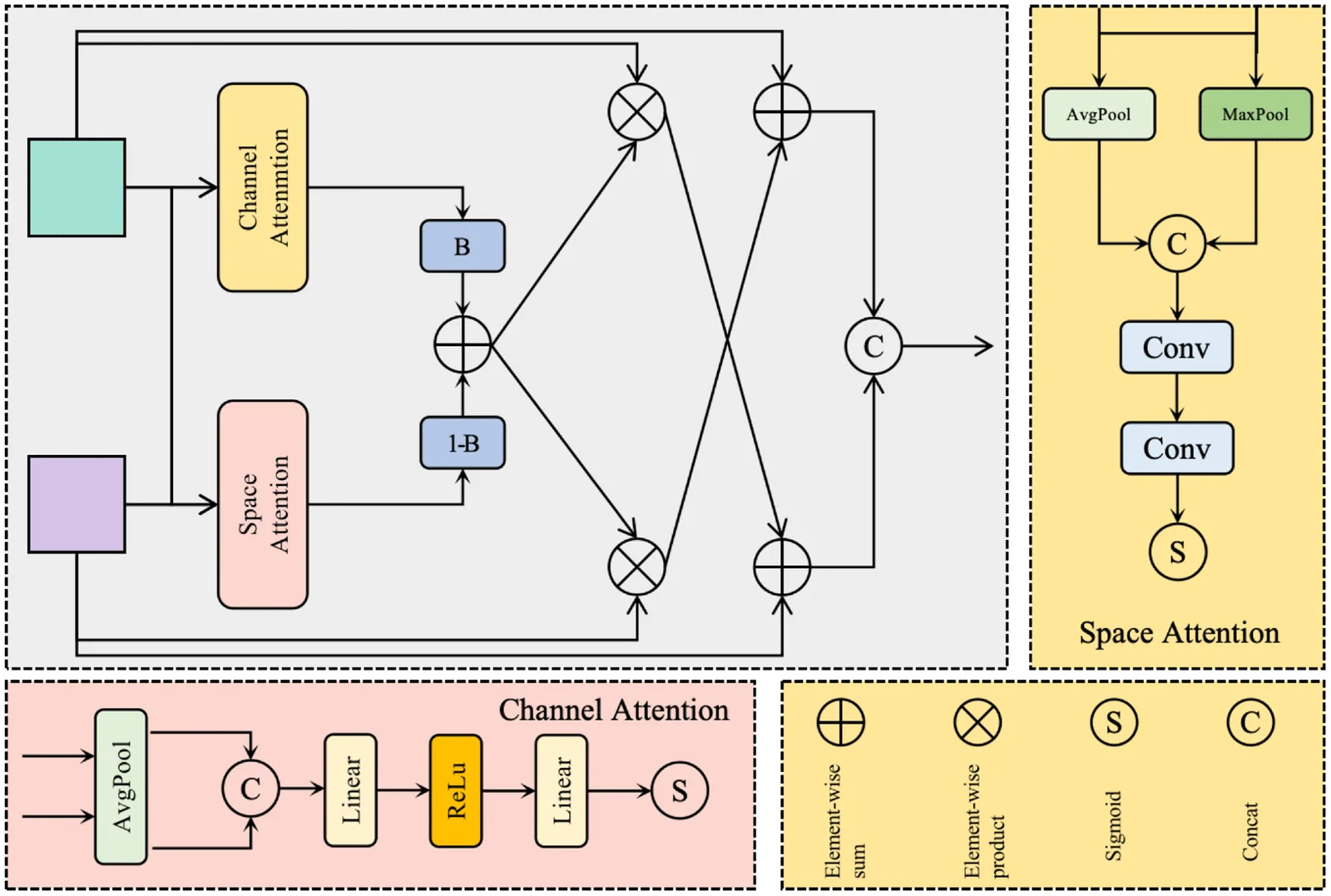
The structure of CAOFM.

Considering that adjacent feature maps provide complementary information, the CAOFM is designed to further enhance the boundary discrimination performed by the FSAM, as shown in Fig 7. Conventional architectures like FPN or PAN rely on top-down or bottom-up propagation. However, indirect information transfer across multiple intermediate layers can cause feature misalignment. This often results in the loss of small and occluded targets during scale transformations. In contrast, the CAOFM emphasizes fully leveraging the strong complementarity among adjacent feature maps instead of focusing on long-range fusion. The CAOFM leverages the extensive overlap of receptive fields across adjacent layers. Through pairwise interactions, the module effectively leverages the complementary information of adjacent feature maps. Consequently, this mechanism maintains the semantic integrity of high-density instances before the prediction stage.

Specifically, the feature maps of different scales are unified to the same spatial dimensions, denoted as $t_{1},t_{2}\in {\mathbb{R}}^{B\times C\times H\times W}$. CAOFM first models the dependency features between channel and spatial attention adaptively. The spatial attention is generated by concatenating the average pooling and max pooling features, as shown in Eq. (9).


$$\begin{array}{c} A_{t_{1}}=mean(t_{1})\in {\mathbb{R}}^{B\times 1\times H\times W},\;M_{t_{1}}=\max(t_{1})\in {\mathbb{R}}^{B\times 1\times H\times W},\;A_{t_{2}}=mean(t_{2})\in {\mathbb{R}}^{B\times 1\times H\times W},\;M_{t_{2}}=\max(t_{2})\in {\mathbb{R}}^{B\times 1\times H\times W},\;S_{att}=\sigma \left(Conv(Conv(Concat[A_{t_{1}},M_{t_{1}},A_{t_{2}},M_{t_{2}}]))\right). \end{array}$$ (9)

The channel attention captures the dependencies between channels through global average pooling and MLP, as shown in Eq. (10).


$$\begin{array}{c} T_{1},T_{2}=GAP(t_{1},t_{2})\in {\mathbb{R}}^{B\times 2C}.\;C_{att}=\sigma (Linear(\delta (Linear(Concat[T_{1},T_{2}])))), \end{array}$$ (10)

where GAP denotes global average pooling, Concat denotes channel-wise concatenation, and Linear represents a fully connected layer. σ and δ denote the Sigmoid and ReLU activation functions, respectively.

Subsequently, the two types of attention are fused using a learnable factor *B* to dynamically balance their contributions. Finally, cross-scale feature interaction is achieved through weighted addition and concatenation operations, as shown in Eq. (11).


$$\begin{array}{c} A=B\cdot C_{att}+(1-B)\cdot S_{att},\;Y=Concat[t_{2}+t_{1}⊙A,\;t_{1}+t_{2}⊙A]. \end{array}$$ (11)

This module effectively fuses complementary information across multiple scales by adaptively integrating spatial and channel-wise attention, thereby mitigating feature misalignment. Additionally, for severely occluded livestock, the module recovers missing feature representations by leveraging information from adjacent scales, thereby enhancing detection performance.

## Experiments

### Experimental setup

In this study, model training was conducted on a server equipped with an NVIDIA GeForce RTX 3090 GPU. The training environment uses CUDA 11.8 and Python 3.10. The AdamW optimizer was used with a default learning rate of 0.0008, weight decay, and a batch size of 8, training for a total of 160 epochs. Three different versions of DOLD-Net were designed: DOLD-Net-N (lightweight version), DOLD-Net-S (small-scale version), and DOLD-Net-M (medium-scale version). These variants differ in model size and computational complexity to suit different application scenarios. Specifically, DOLD-Net-N is designed for resource-constrained environments, DOLD-Net-S achieves a balance between model accuracy and size, and the DOLD-Net-M is suitable for scenarios with higher accuracy requirements. The Average Precision (AP) metric from the COCO evaluation framework [27] was chosen as the primary performance evaluation metric in this study.

### Experimental results

Table 1 presents a performance comparison of different models on the $GooseDetect_{b}^{lion}$ dataset. Compared with D-FINE, the proposed DOLD-Net improves *AP* by 1.0%, 1.2%, and 1.8% for the N, S, and M variants, respectively. It also reduces the number of parameters by 8%, 16%, and 13%, and lowers the computational cost by 2%, 10%, and 7%. These results indicate that DOLD-Net substantially reduces model complexity while enhancing detection performance. Comparison with the YOLO series, DOLD-Net also demonstrates strong performance. Specifically, DOLD-Net-N achieves a 2.0% *AP* improvement over YOLOv8 while reducing computational cost by 21%. DOLD-Net-S improves *AP* by 3.4% compared with YOLOv9-S and reduces computational cost by 17%. DOLD-Net-M achieves a 2.0% higher *AP* than YOLOv12-M while reducing computational cost by 23% and the number of parameters by 18%. This improvement can be attributed to the CGFP module, which minimizes computational overhead while enhancing detection capability for densely occluded livestock. These results further validate the effectiveness of DOLD-Net for dense occlusion livestock detection.

**Table 1:** Performance comparison of different models on the $GooseDetect_{b}^{lion}$ dataset.

Method	*AP*	*AP*_50_	*AP*_75_	FLOPs	Params
YOLOv8-N [28]	50.4	85.8	53.2	8.7G	3.2M
YOLOv8-S [28]	53.6	87.7	58.2	28.6G	11.2M
YOLOv8-M [28]	55.0	88.7	60.3	78.9G	25.9M
YOLOv8-L [28]	55.6	88.7	60.5	165.4G	43.6M
YOLOv9-T [29]	49.2	83.9	51.9	7.7G	2.0M
YOLOv9-S [29]	54.1	87.9	58.4	26.7G	7.2M
YOLOv9-M [29]	55.6	89.0	60.7	76.8G	20.1M
YOLOv9-C [29]	55.5	88.9	60.3	103.7G	25.5M
YOLOv10-N [10]	49.5	84.0	52.3	8.4G	2.7M
YOLOv10-S [10]	53.0	87.2	56.9	24.8G	8.6M
YOLOv10-M [10]	54.9	88.5	59.9	58.9G	15.3M
YOLOv10-L [10]	55.4	88.8	60.3	127.2G	25.7M
YOLOv11-N [30]	50.2	85.0	52.8	6.4G	2.6M
YOLOv11-S [30]	53.9	87.6	58.2	21.5G	9.4M
YOLOv11-M [30]	55.7	88.9	60.8	68.0G	20.1M
YOLOv11-L [30]	56.2	89.0	61.4	87.3G	25.3M
YOLOv12-N [31]	49.1	84.6	50.8	6.5G	2.5M
YOLOv12-S [31]	53.2	87.3	57.0	21.5G	9.2M
YOLOv12-M [31]	55.7	88.9	60.8	67.5G	20.2M
YOLOv12-L [31]	55.9	88.9	61.2	88.5G	26.3M
RT-DETR-R18 [32]	52.0	86.0	55.9	59.8G	19.8M
RT-DETR-R34 [32]	55.5	88.6	60.2	91.9G	31.1M
D-FINE-N [18]	51.4	86.5	54.3	7.1G	3.7M
D-FINE-S [18]	56.3	89.0	61.9	24.8G	10.1M
D-FINE-M [18]	55.9	88.9	61.1	56.3G	19.1M
DEIM-N [33]	49.0	83.8	51.7	7.1G	3.7M
DEIM-S [33]	55.2	88.1	60.6	24.8G	10.1M
DEIM-M [33]	53.6	87.5	57.9	56.3G	19.1M
**DOLD-Net-N (Ours)**	**52.4(+3.4)**	**86.9**	**55.7**	6.9G	3.4M
**DOLD-Net-S (Ours)**	**57.5(+2.3)**	**89.5**	**63.4**	22.3G	8.4M
**DOLD-Net-M (Ours)**	**57.7(+4.1)**	**90.2**	**63.2**	52.2G	16.6M

The values in parentheses indicate the improvements of DOLD-Net over the DEIM model with the same scale, and the same convention applies hereafter.

Table 2 presents the quantitative results on the $GooseDetect_{s}^{lion}$ subset, while Table 3 illustrates the model performance on the CherryChèvre dataset. DOLD-Net achieves outstanding performance on both datasets. Specifically, DOLD-Net-N, DOLD-Net-S, and DOLD-Net-M achieve *AP* improvements of 1.2%, 3.9%, and 7.2% over DEIM on the $GooseDetect_{s}^{lion}$ subset, respectively.

**Table 2:** Performance comparison of different models on the $GooseDetect_{s}^{lion}$ dataset.

Method	*AP*	*AP*_50_	*AP*_75_
YOLOv11-N [30]	39.6	80.3	33.7
YOLOv11-S [30]	42.6	83.8	38.4
YOLOv11-M [30]	44.1	84.2	40.7
YOLOv12-N [31]	39.0	79.8	32.3
YOLOv12-S [31]	42.7	83.5	37.4
YOLOv12-M [31]	43.8	84.3	40.0
YOLOv12-L [31]	44.5	86.7	40.4
RT-DETR-R18 [32]	44.7	84.7	41.7
RT-DETR-R34 [32]	44.1	83.5	40.9
D-FINE-N [18]	35.9	76.0	25.0
D-FINE-S [18]	43.1	82.5	39.3
D-FINE-M [18]	41.9	82.5	37.3
DEIM-N [33]	39.8	80.4	33.4
DEIM-S [33]	43.0	83.9	38.3
DEIM-M [33]	40.4	79.6	35.8
**DOLD-Net-N (Ours)**	**41.0(+1.2)**	**81.7**	**35.5**
**DOLD-Net-S (Ours)**	**46.9(+3.9)**	**89.0**	**46.1**
**DOLD-Net-M (Ours)**	**47.6(+7.2)**	**86.8**	**47.6**

**Table 3:** Performance comparison of different models on the CherryChèvre dataset.

Method	*AP*	*AP*_50_	*AP*_75_
YOLOv11-N [30]	56.3	86.4	63.0
YOLOv11-S [30]	59.6	89.1	66.5
YOLOv11-M [30]	62.1	90.2	69.8
YOLOv12-N [31]	55.9	86.2	62.5
YOLOv12-S [31]	59.4	87.9	67.3
YOLOv12-M [31]	61.7	89.6	69.6
YOLOv12-L [31]	62.0	90.0	69.6
RT-DETR-R18 [32]	65.4	94.1	73.7
D-FINE-N [18]	57.4	88.1	62.3
D-FINE-S [18]	64.2	93.0	72.2
D-FINE-M [18]	63.5	92.9	70.8
DEIM-N [33]	60.6	91.0	66.6
DEIM-S [33]	65.9	93.9	74.4
DEIM-M [33]	65.9	94.1	74.7
**DOLD-Net-N (Ours)**	**62.6(+2.0)**	**92.3**	**69.3**
**DOLD-Net-S (Ours)**	**67.1(+1.2)**	**95.9**	**75.6**
**DOLD-Net-M (Ours)**	**67.5(+1.6)**	**96.7**	**76.3**

Table 4 reports the performance comparison of different models on the SheepCounter dataset, while Table 5 presents the corresponding results on the ChickenFlow dataset. DOLD-Net achieves outstanding performance on both datasets. Specifically, on the SheepCounter dataset, DOLD-Net-N, DOLD-Net-S, and DOLD-Net-M achieve *AP* gains of 0.8%, 0.5%, and 1.2% over DEIM, respectively. On the ChickenFlow dataset, DOLD-Net-N and DOLD-Net-S achieve *AP* improvements of 0.8% over DEIM. Overall, these experimental results demonstrate the strong generalization ability and robustness of DOLD-Net across different livestock species, occlusion levels, and environmental conditions, highlighting its effectiveness in a wide range of livestock detection tasks.

**Table 4:** Performance comparison of different models on the SheepCounter dataset.

Method	*AP*	*AP*_50_	*AP*_75_
YOLOv11-N [30]	58.0	96.4	63.6
YOLOv11-S [30]	58.8	96.6	66.3
YOLOv11-M [30]	59.6	96.7	67.9
YOLOv12-N [31]	58.0	96.5	63.7
YOLOv12-S [31]	58.8	96.6	65.7
YOLOv12-M [31]	59.0	96.6	66.6
YOLOv12-L [31]	59.1	96.7	66.4
RT-DETR-R18 [32]	59.8	96.6	67.8
RT-DETR-R50 [32]	58.1	96.4	64.3
D-FINE-N [18]	58.4	96.4	64.8
D-FINE-S [18]	60.8	97.3	69.8
D-FINE-M [18]	60.1	96.9	68.1
DEIM-N [33]	59.4	96.6	67.8
DEIM-S [33]	61.7	97.5	71.6
DEIM-M [33]	61.3	97.4	70.4
**DOLD-Net-N (Ours)**	**60.2(+0.8)**	**96.9**	**68.5**
**DOLD-Net-S (Ours)**	**62.2(+0.5)**	**97.6**	**72.4**
**DOLD-Net-M (Ours)**	**62.5(+1.2)**	**97.7**	**72.7**

**Table 5:** Performance comparison of different models on the ChickenFlow dataset.

Method	*AP*	*AP*_50_	*AP*_75_
Faster R-CNN-R50 [34]	37.7	71.2	34.1
Faster R-CNN-R101 [34]	40.1	73.3	37.4
YOLOv11-N [30]	50.9	79.4	53.8
YOLOv11-S [30]	51.4	78.8	54.3
YOLOv11-M [30]	51.1	78.7	53.9
YOLOv11-L [30]	50.6	79.0	53.6
YOLOv12-N [31]	50.2	78.8	53.0
YOLOv12-S [31]	51.7	79.6	**55.3**
YOLOv12-M [31]	51.1	78.7	53.9
YOLOv12-L [31]	51.1	78.9	53.5
RTMDet-T [35]	51.5	79.3	55.1
RTMDet-S [35]	51.2	78.9	54.9
RT-DETR-R18 [32]	50.4	77.5	53.1
RT-DETR-R34 [32]	51.4	78.1	54.1
RT-DETR-R50 [32]	51.5	78.2	54.6
D-FINE-N [18]	51.0	77.8	53.8
D-FINE-S [18]	51.4	77.4	54.5
D-FINE-M [18]	51.6	78.0	54.9
DEIM-N [33]	51.2	78.1	54.2
DEIM-S [33]	51.4	78.2	54.4
DEIM-M [33]	51.7	78.2	55.1
**DOLD-Net-N (Ours)**	**52.0(+0.8)**	**79.5**	**55.3**
**DOLD-Net-S (Ours)**	**52.2(+0.8)**	**79.8**	55.0

### Ablation experiment

To validate the effectiveness of each module proposed in this paper for livestock detection under dense occlusion, this section conducts detailed ablation experiments on each module. Furthermore, a detailed analysis and comparison of the design components within each module are provided. Table 6 presents the ablation results of the individual modules across different datasets. First, the Dual-Branch Occlusion-Aware Network (DBOAN) improves AP across all datasets consistently. This suggests that local convolutions are inadequate for processing densely distributed targets during the early stages of feature extraction. In contrast, the dual-branch mechanism establishes a robust feature representation that incorporates global topological information. Notably, these improvements are achieved with only a negligible increase in the number of parameters. Moreover, the Context-Guided Focus Propagation (CGFP) framework improves AP and decreases both parameter count and computational cost. These results indicate that constructing a global semantic prior effectively compensates for missing information in multi-scale features while improving efficiency.

**Table 6:** Ablation results of different modules across various datasets.

Dataset	DBOAN	CGFP	GFLOPs	Param	*AP*
$GooseDetect_{b}^{lion}$			7.1G	3.7M	49.0
	✓		7.1G	3.8M	50.4
		✓	6.8G	3.4M	52.0
	✓	✓	6.9G	3.4M	**52.4**
$GooseDetect_{s}^{lion}$			7.1G	3.7M	39.8
	✓		7.1G	3.8M	40.1
		✓	6.8G	3.4M	40.4
	✓	✓	6.9G	3.4M	**41.0**
ChickenFlow			7.1G	3.7M	51.2
	✓		7.1G	3.8M	51.7
		✓	6.8G	3.4M	51.7
	✓	✓	6.9G	3.4M	**52.4**
CherryChèvre			7.1G	3.7M	60.6
	✓		7.1G	3.8M	61.8
		✓	6.8G	3.4M	62.0
	✓	✓	6.9G	3.4M	**62.6**
SheepCounter			7.1G	3.7M	59.4
	✓		7.1G	3.8M	59.6
		✓	6.8G	3.4M	59.7
	✓	✓	6.9G	3.4M	**60.2**

To further investigate the effectiveness of the individual components within the CGFP module, a systematic ablation study was conducted (Table 7). The results demonstrate that the Cross-Scale Feature Enhancer (CSFE) module plays a primary role in enhancing detection precision; specifically, the *AP* increased from 49.0% to 51.1% on the $GooseDetect_{b}^{lion}$ dataset and by 0.6% on the CherryChèvre dataset. This improvement is attributed to the integration of global semantic priors via the CSFE, which compensates for the limitations of local feature representation and enhances model robustness in complex scenarios. Notably, the model achieves optimal performance when CSFE, FSAM, and CAOFM are integrated, validating the complementarity and synergistic potential of the sub-modules in boosting overall detection capability.

**Table 7:** Ablation results of different CGFP module components.

Dataset	CSFE	FSAM	CAOFM	*AP*
$GooseDetect_{b}^{lion}$				49.0
	✓			51.1
		✓		50.3
			✓	50.4
	✓	✓		51.6
	✓		✓	51.4
		✓	✓	50.9
	✓	✓	✓	**52.0**
CherryChèvre				60.6
	✓			61.2
		✓		60.8
			✓	61.0
	✓	✓		61.5
	✓		✓	61.7
		✓	✓	61.2
	✓	✓	✓	**62.0**

To investigate the impact of kernel sizes within the CSFE module on model performance, this paper conducted ablation experiments as shown in Table 8. The experimental results demonstrate that using fixed-size kernel sequences leads to varying degrees of performance degradation compared to the multi-scale kernel combination (5, 7, 9, 11). Single-scale kernels struggle to capture cross-scale spatial information within feature maps. This shortcoming limits the construction of global core priors. Ultimately, it weakens the model’s capacity to leverage global context for effective object detection.

**Table 8:** Ablation results of different kernel size settings.

Kernel Size	*AP*	*AP*_50_	*AP*_75_
(3,3,3,3)	51.7	86.9	54.6
(5,5,5,5)	51.9	86.3	55.1
(7,7,7,7)	51.5	86.2	54.8
(5,7,9,11)	**52.4**	**86.9**	**55.7**

To further investigate the impact of different propagation counts and locations on model performance in CGFP, an ablation study of the propagation strategy was conducted. Table 9 presents the effects of varying propagation counts and locations on model performance. Specifically, “First Propagation” refers to the propagation operation applied after the backbone, “Second Propagation” denotes the propagation operation inserted after the CAOFM module. The results show that both propagation stages effectively improve model performance on the SheepCounter and $GooseDetect_{b}^{lion}$ datasets. This improvement arises from the first propagation, which injects global contextual information into multi-scale feature maps. Consequently, the model better identifies targets under densely occluded conditions. In contrast, the second propagation further refines the feature representations derived from the first stage. This demonstrates the effectiveness of multi-stage propagation in capturing and exploiting global contextual information. Furthermore, the impact of employing three propagation stages on model performance was also examined. However, the experimental results indicate that increasing the number of propagations does not lead to significant improvement. In fact, it may lead to performance stagnation or even degradation. This phenomenon stems from redundant feature accumulation due to excessive propagation, which degrades learning efficiency and generalization. In conclusion, the proposed dual-propagation mechanism demonstrates clear advantages in dense occlusion livestock detection tasks, improving overall detection performance effectively.

**Table 9:** Impact of different propagation counts and locations on model performance.

First Propagation	Second Propagation	*AP*
SheepCounter		
✓		59.8
	✓	59.3
✓	✓	**60.2**
$GooseDetect_{b}^{lion}$		
✓		52.3
	✓	51.7
✓	✓	**52.4**

**Note:** ✓ indicates that the corresponding propagation operation is applied. Results are reported under identical training settings.

To verify the effectiveness of the proposed Context-Aware Occlusion Fusion Module (CAOFM) in handling dense occlusion, this study compares CAOFM with several alternative fusion strategies. The performance of each fusion method is presented in Table 10. The results show that CAOFM outperforms all other fusion methods, demonstrating its effectiveness in capturing complex features under densely occluded conditions. These experiments further confirm that the proposed fusion approach improves model performance, particularly in scenarios involving severe occlusion.

**Table 10:** Impact of different fusion methods on the performance of the model.

Method	*AP*	GFLOPs	Param
DPCF [36]	51.2	6.0G	3.4M
MFM [37]	51.6	6.5G	3.4M
MPCA [38]	50.9	7.0G	3.6M
WFU [39]	51.9	8.1G	4.2M
**CAOFM (Ours)**	**52.4**	6.9G	3.4M

Moreover, to verify the effectiveness of the proposed Frequency-Spatial Anti-occlusion Module (FSAM) module in addressing dense occlusion, this study conducts comparative experiments between FSAM and several commonly used feature extraction modules. Table 11 compares the performance of different feature extraction modules. The comparison results show that the FSAM module has a clear advantage in extracting information related to occluded regions, especially under dense occlusion. Under such conditions, it captures critical information from occluded targets more effectively. The global–local feature extraction mechanism of FSAM enhances the model’s ability to distinguish objects in complex environments. This leads to improved performance on densely occluded detection tasks.

**Table 11:** Impact of different feature extraction modules on the performance of the model.

Method	*AP*	GFLOPs	Param
RepViTBlock [40]	52.0	6.6G	3.4M
DBlock [41]	51.7	6.4G	3.3M
C3K2 [30]	51.0	6.3G	3.2M
C2F [28]	51.9	6.9G	3.4M
MRFFI [42]	51.3	6.3G	3.3M
**FSAM (Ours)**	**52.4**	6.9G	3.4M

To further justify the choice of the Haar wavelet in the FSAM module, we compared it with several higher-order orthogonal wavelets, including db2, db4, and db8, on the $GooseDetect_{s}^{lion}$ dataset. As shown in Table 12, the Haar wavelet achieves the best detection performance with the lowest computational cost. Owing to its compact support, Haar provides accurate spatial localization and preserves sharp object boundaries. In contrast, higher-order Daubechies wavelets have broader support regions. This often causes excessive smoothing and feature leakage between neighboring instances in densely populated scenes. Consequently, the overall performance degrades. Among them, db4 performs better than db2 and db8 because its increased vanishing moments enhance local texture representation and partially alleviate boundary blurring. However, the larger support width of db8 leads to more severe inter-object feature contamination, outweighing the benefits of its richer frequency representation and consequently reducing detection accuracy.

**Table 12:** Comparison of different wavelet decomposition methods in the FSAM module.

Wavelet Type	*AP*	*AP*_50_	*AP*_75_	GFLOPs
**Haar (db1)**	**41.0**	**81.7**	**35.5**	**+0.0G**
db2	40.2	81.1	35.3	+0.15G
db4	40.7	81.6	35.3	+0.17G
db8	40.3	81.4	35.2	+0.21G

To assess the core CSFE operator, VSSM was replaced with the Transformer’s Multi-Head Self-Attention (MHSA) module. As shown in Table 13, the Transformer variant exhibited a performance decline. The experiment indicates that they remain susceptible to interference from background noise in dense scenarios. In contrast, VSSM effectively highlights target regions and mitigates feature loss caused by occlusion via dynamic context modeling. Furthermore, the Transformer still surpasses other comparative models, thereby validating the effectiveness of the proposed global core feature construction and propagation mechanism.

**Table 13:** Impact of different core operators within the CSFE module on model performance.

Core Operator	*AP*	FLOPs	Params
MHSA	52.0	7.0G	3.5M
**VSSM**	**52.4**	6.9G	3.4M

### Visualization analysis

**Fig 8:**
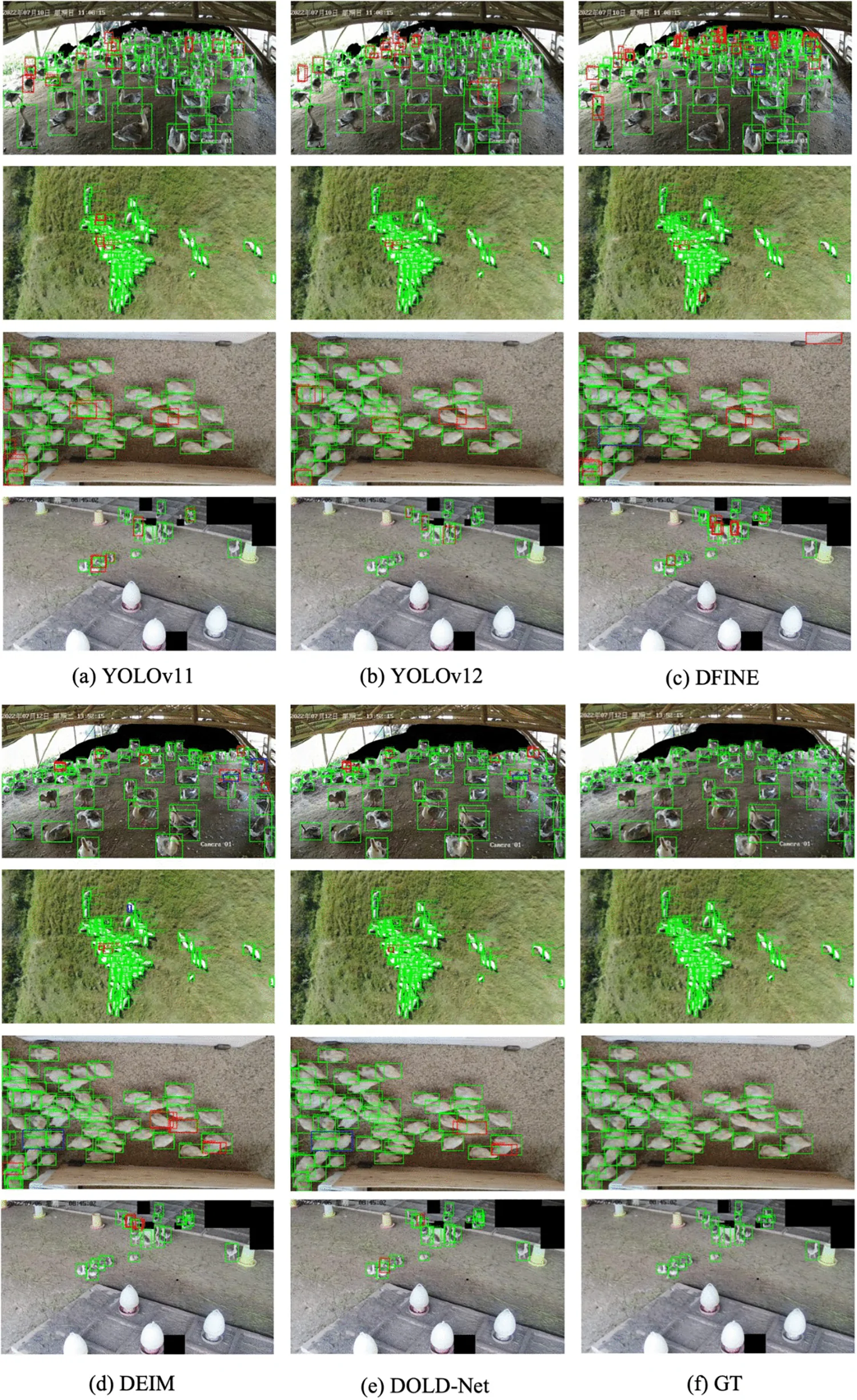
Visual results of different models on the task of livestock detection under dense occlusion. Green boxes denote correctly detected targets (TP), red boxes denote falsely detected targets (FP), and blue boxes denote missed targets (FN). Images in rows 1, 4, 5 and 8 sourced from [19], available under the CC0 public domain dedication. Images in rows 2 and 6 sourced from [43] under the CC BY 4.0 license. Images in rows 3 and 7 sourced from [3] under the CC BY 4.0 license.

To evaluate detection performance for densely occluded livestock qualitatively, Fig 8 shows the results of YOLO series models and DETR-based models on different datasets. It can be observed that all models are able to accurately detect the targets, whereas in densely occluded areas, a large number of missed and false detections occur. Compared with existing methods, DOLD-Net achieves the lowest miss and false detection rates. The remaining false detections mainly occur in regions where object boundaries are ambiguous and heavily occluded. This indicates that the proposed method is overall significantly superior to existing state-of-the-art methods. It also demonstrates strong capability in livestock detection under dense occlusion.

**Fig 9:**
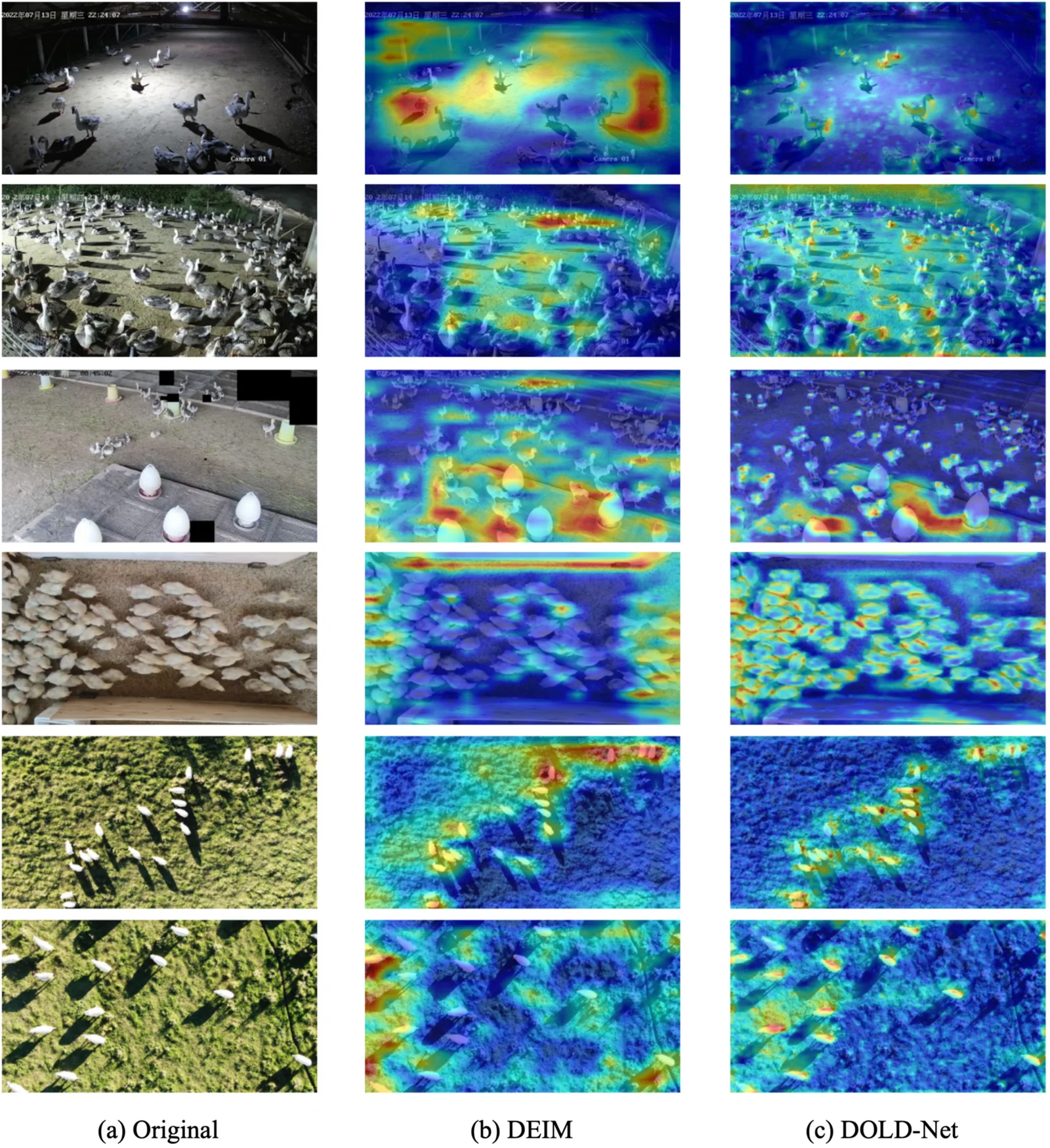
Heatmap across different datasets. Warmer colors indicate stronger model responses or attention, whereas cooler colors indicate weaker responses. Images in rows 1, 2 and 3 sourced from [19], available under the CC0 public domain dedication. Images in rows 4 sourced from [3] under a CC BY 4.0 license. Images in rows 5 and 6 sourced from [43] under the CC BY 4.0 license.

This study employs heatmaps to perform a visual analysis of the model’s attention regions. Fig 9 compares the original images with heatmaps generated by the DEIM and DOLD-Net models across different datasets. The heatmaps of DOLD-Net show concentrated activations within target regions, particularly on discriminative areas such as the heads of geese. This focus enables DOLD-Net to more effectively distinguish individual animals under dense occlusion. In contrast, the heatmaps of the DEIM model exhibit more dispersed activations within the target regions. It also fails to maintain sufficient focus on certain individuals. As a result, this limitation may lead to missed detections or false positives. Overall, the heatmap analysis further validates the advantages of DOLD-Net for livestock detection under dense occlusion. By focusing on critical discriminative regions, the model effectively enhances detection performance.

**Fig 10:**
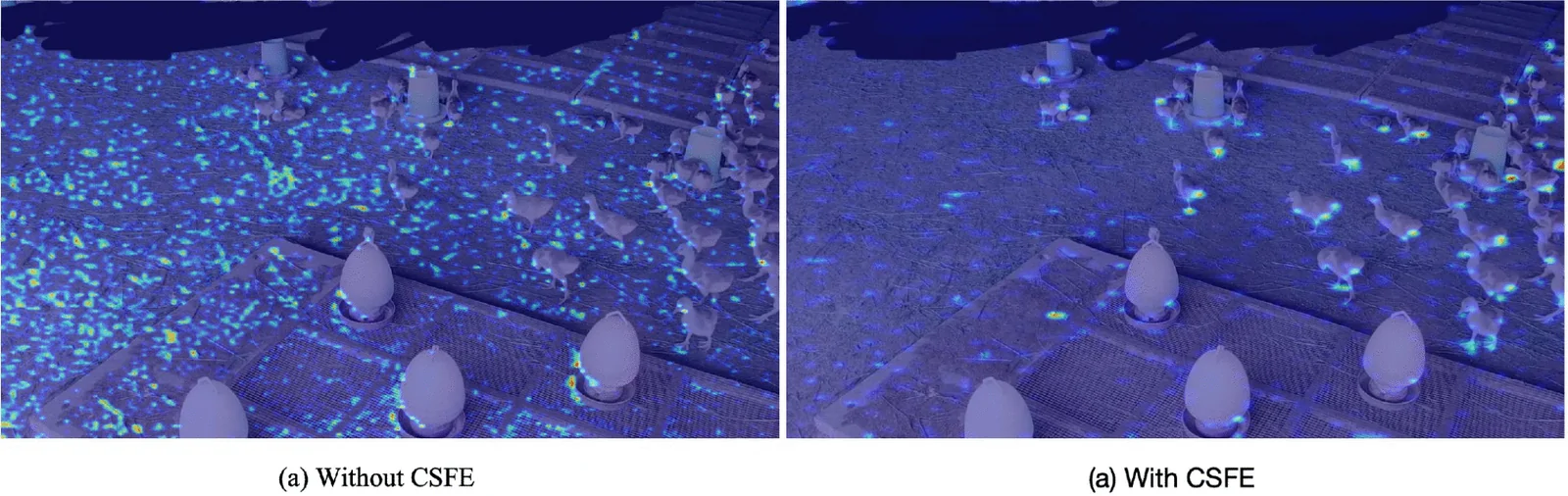
Feature map heatmaps with and without CSFE. Image sourced from [19], available under the CC0 public domain dedication.

Moreover, to assess the effectiveness of the global representations learned by the proposed CSFE in dense-occlusion livestock detection, a heatmap-based comparison is performed. Feature maps generated with and without CSFE are compared, as shown in Fig 10. The model without CSFE attends to target regions but suffers from substantial noise, which hinders accurate detection. In contrast, the model with CSFE can effectively guide the network to focus on target regions using the encoded global contextual information. It also significantly suppresses background noise. Consequently, the model is able to learn the feature representations of individual animals more accurately. This further validates the effectiveness of CSFE in improving performance on dense-occlusion livestock detection tasks.

## Conclusions and discussion

This study proposes DOLD-Net, an efficient detection framework. The framework is tailored to tackle the challenges of high density, severe occlusion, and ambiguous boundaries in livestock monitoring. DOLD-Net adopts a “global-local” feature collaboration paradigm to systematically reduce feature degradation in complex environments. Specifically, the Dual-Branch Occlusion-Aware Network (DBOAN) serves as a robust backbone by jointly modeling local gradient flows and global topological structures to separate adhered individuals.Furthermore, the Context-Guided Focus Propagation (CGFP) mechanism leverages the VSSM to construct global semantic priors, inferring occluded regions via top-down propagation. To resolve feature entanglement, the Frequency-Spatial Anti-occlusion Module(FSAM) decouples features in the frequency domain to sharpen blurred boundaries. Meanwhile, the Context-Aware Occlusion Fusion Module (CAOFM) bridges semantic gaps by exploiting cross-scale complementarity.

Extensive experiments on four public datasets demonstrate that DOLD-Net achieves a superior balance between accuracy, parameter efficiency, and computational cost. Compared with state-of-the-art methods such as YOLO-v12 and D-FINE, DOLD-Net exhibits stronger robustness and generalization capabilities.

**Fig 11:**
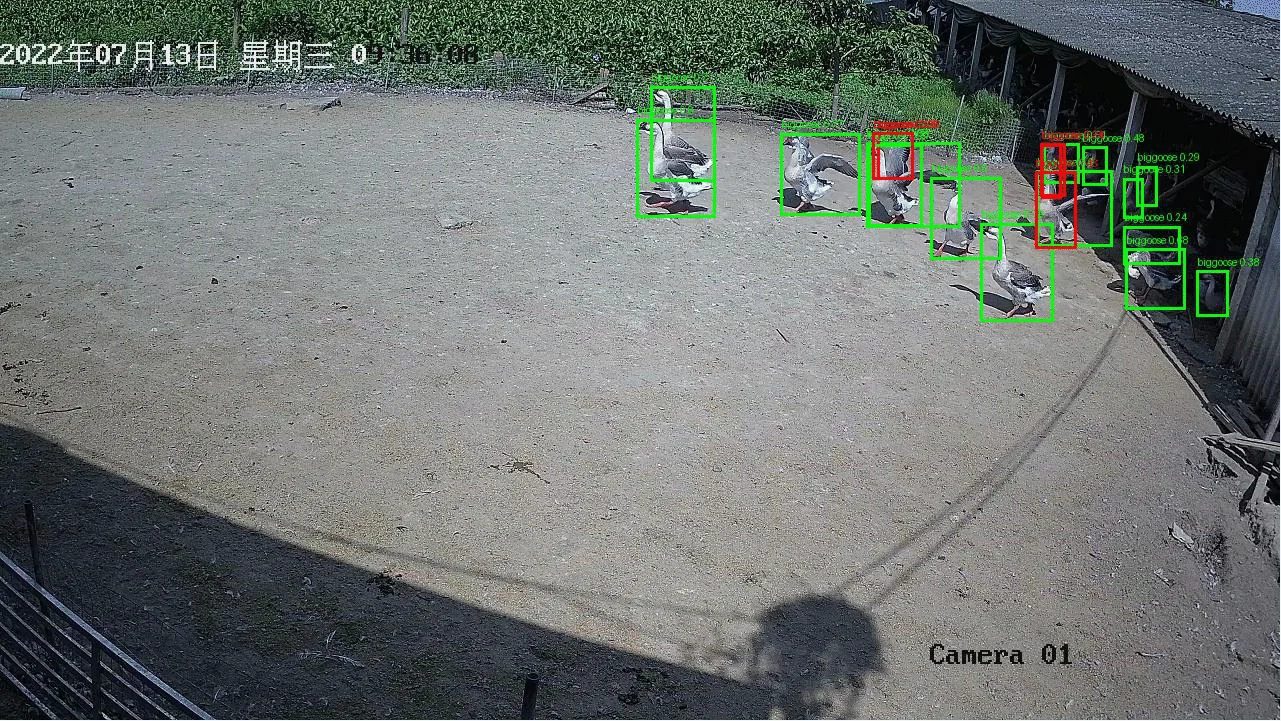
Failure case of DOLD-Net under complex pose conditions. Image sourced from [19], available under the CC0 public domain dedication.

Although the proposed model achieves promising detection performance, several limitations remain. Large pose variations pose a challenge to the model and can adversely affect feature representation. Consequently, false positive predictions may occur, as shown in Fig 11. The goose undergoes large-amplitude wing movements. This results in substantial appearance changes in the upper-right region. Such variations mislead the model and cause the moving wing area to be mistakenly detected as a separate goose instance. Extreme pose variations disrupt the original global topological structure of livestock targets. This disruption causes them to deviate from the typical shape distribution learned by the model during training. SSM captures long-range dependencies by recursively propagating historical information through the hidden state $h_{t}$. Under regular livestock poses, semantic sequences generated by different scanning directions exhibit high semantic consistency. Therefore, SS2D can effectively learn stable state-transition patterns and establish a global semantic prior.

**Fig 12:**
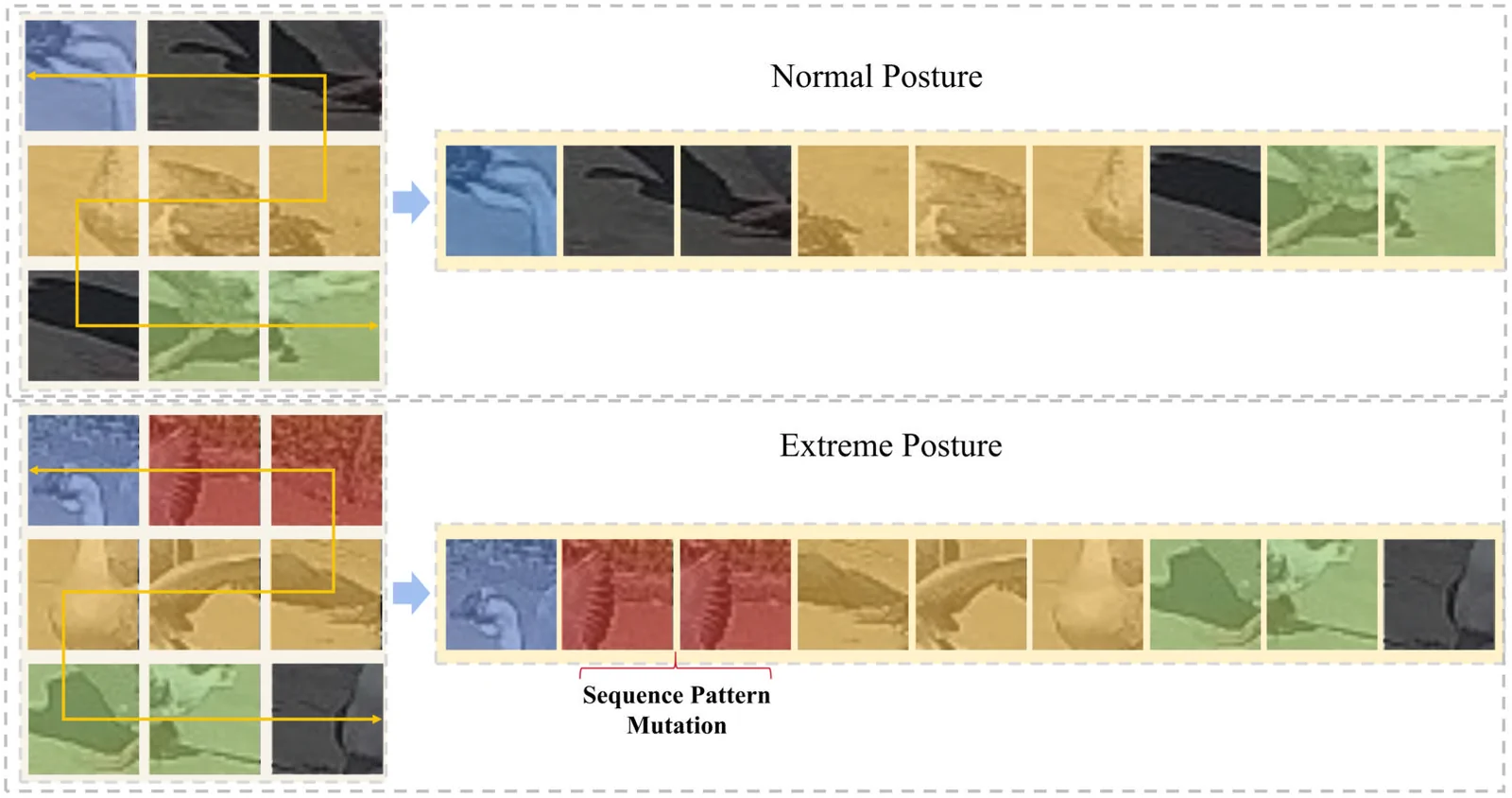
Comparison of 1D Semantic Sequence Unrolling between Normal and Extreme Poses. Image sourced from [19], available under the CC0 public domain dedication.

However, extreme pose variations introduce significant non-rigid deformations to the target’s spatial structure. As shown in Fig 12, SS2D learns stable state-transition patterns from the semantic order of the head, torso, and limbs under normal poses. Large pose changes disrupt this semantic order, causing a mismatch between the learned transitions and the input sequence. As a result, the CSFE module fails to effectively aggregate global contextual information, weakening global semantic modeling and leading to erroneous predictions.

Future work targets two directions: optimizing model inference efficiency and embedding skeletal geometric priors to model inter-instance topological correlations. Training corpus will be augmented with extreme pose deformation samples to enrich intra-class diversity, strengthen out-of-distribution robustness, and boost generalization against severe morphological perturbations.
